# Research and Development of Hepatitis B Drugs: An Analysis Based on Technology Flows Measured by Patent Citations

**DOI:** 10.1371/journal.pone.0164328

**Published:** 2016-10-11

**Authors:** Chuoji Huang, Hui Heng Lin, Jian-bo Wan, Chengwei He, Yuanjia Hu

**Affiliations:** State Key Laboratory of Quality Research in Chinese Medicine, Institute of Chinese Medical Sciences, University of Macau, Avenida da Universidade, Taipa, Macau SAR, 999078, China; National Chiao Tung University College of Biological Science and Technology, TAIWAN

## Abstract

Despite the existence of available therapies, the Hepatitis B virus infection continues to be one of the most serious threats to human health, especially in developing countries such as China and India. To shed light on the improvement of current therapies and development of novel anti-HBV drugs, we thoroughly investigated 212 US patents of anti-HBV drugs and analyzed the technology flow in research and development of anti-HBV drugs based on data from IMS LifeCycle databases. Moreover, utilizing the patent citation method, which is an effective indicator of technology flow, we constructed patent citation network models and performed network analysis in order to reveal the features of different technology clusters. As a result, we identified the stagnant status of anti-HBV drug development and pointed the way for development of domestic pharmaceuticals in developing countries. We also discussed about therapeutic vaccines as the potential next generation therapy for HBV infection. Lastly, we depicted the cooperation between entities and found that novel forms of cooperation added diversity to the conventional form of cooperation within the pharmaceutical industry. In summary, our study provides inspiring insights for investors, policy makers, researchers, and other readers interested in anti-HBV drug development.

## Introduction

The hepatitis B virus (HBV) is the cause of one of the most common viral infections in the world [1]. HBV spreads primarily through transcutaneous or mucosal exposure to blood or other body fluids from infected hosts [2]. A number of studies have shown that active HBV replication leads to liver injury and disease progression [3]. Patients with chronic HBV infection suffer from risks of liver fibrosis, cirrhosis, and hepatocellular carcinoma (HCC) and may eventually die from liver failure or other complications [4]. According to the World Health Organization (WHO), two billion people have been infected with HBV so far and 240 million people worldwide were chronic carriers of HBV surface antigen (HBsAg) by the end of 2014 [1]. Though prophylactic vaccines for HBV have been available for over 30 years thanks to universal hepatitis B immunization programs, these preventive vaccines are not enough in protecting infected population from HBV-related deaths, especially those who have been infected prior to the launch of the program [1]. As a result, around 650,000 people die each year from the complications of chronic hepatitis B (CHB) [1, 5].

Currently, available treatments for chronic hepatitis B depend primarily on nucleoside analogs (NAs), which effectively inhibit virus replication but fail to eliminate the virus. Consequently, NAs merely prolong survival by preventing hepatic decompensation and slowing progression to cirrhosis or HCC [6]. Also, adopting lifelong therapies is typically not an option for patients in developing countries where the infected populations are larger [7, 8]. Thus, current treatments against HBV infection are far from satisfactory, and there is an emerging call to develop new therapies that not only improve efficacy and tolerability but also decrease side effects and shorten treatment periods.

Nevertheless, developing new drugs is a difficult and complex project. Fortunately, an overview of the evolution process of pharmaceutical technologies can provide both guidance for and insights into drug development, including anti-HBV drug development. Cox et al. reviewed the treatments of chronic HBV infection by analyzing the latest safety and efficacy data on existing and emerging agents [9], while our study sheds light on the evolution process of pharmaceutical technologies from a different perspective and approach, i.e., analysis of patent citation networks. Patent citation has been considered an effective representation of knowledge diffusion and has been used to drive innovation [10]. It is important for drug discovery, including new anti-HBV drugs, because all drugs are developed step by step through pharmaceutical technology processes [11–13]. The basic principle of patent citation analysis is based on the theory that citing patents adopt knowledge elements from the patents cited, allowing the evolution process of technological innovations to be modeled as networks [10]; thus, we are able to use patent citation network analysis to obtain valuable insights into the technological development of anti-HBV drugs and the flow of that technology.

So far, to our knowledge, there has not been any report about patent citation network analysis of anti-HBV drugs. In order to fill this gap in the research, we performed a patent citation network analysis of US patents issued for HBV drug development to identify both core and emerging technologies. The purposes of this study are three-fold: First, to illustrate the technology flows of anti-HBV drug research and development (R&D) through patent citation network; second, to characterize the technology communities via cluster comparison in the network models; and third, to provide further insights and advice for investors, pharmaceutical companies, policymakers in governmental organizations, and researchers interested in anti-HBV drug development.

## Methodology

### Research framework

**Fig 1:**
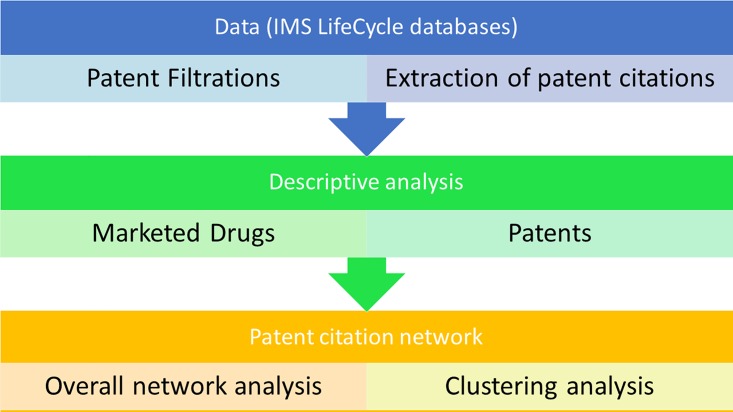
The research framework of this study. Note: Data were collected from IMS LifeCycle databases and processed for statistical analysis, network modeling, and network analysis.

The research framework of this study generally followed a pipeline of database survey (IMS LifeCycle), patent information analysis, and patent citation network analysis, as shown in Fig 1.

We initially performed a systematic database survey across IMS LifeCycle databases, followed by filtering, transform, and integration of patent information. Upon retrieval of the unified data in US patent formats, statistical analysis was performed and network models were built. Finally, we analyzed the data in detail and summarized the conclusions of our study.

### Data

This study collected data from the IMS LifeCycle databases, which is a collection of multi-functional databases about pharmaceutical. IMS R&D Focus covers facets of global drug development, from the discovery phase to availability on the market. IMS Patent Focus is a database providing information on the most significant pharmaceutical patents. Information including estimated patent granted and expiry dates, patent extension information, patent numbers, and originators of marketed compounds, both pharmaceutical and biotechnological, can be found in the databases.

The original patents were identified through a hepatitis B query against IMS databases and listed according to their country of origin. All of patents were transformed into the corresponding US patent format via the patent family system of the European Patent Office (EPO) because analyzing and comparing patent data using one single patent system results in more standard, comparable, and unified patent citation information. Finally, 212 US patents were retrieved and their citations and related information were collected from the IMS databases and the United States Patent and Trademark Office (USPTO) database. We also provided the relevant patent data for public access. They could be found in Supporting Information file(s).

### Descriptive analysis on marketed drugs and patents

In this research, we initially collected patent information about marketed HBV drugs from the IMS Patent Focus database. Patents obtained were manually divided into three categories, i.e., nucleoside analogue, interferon, and vaccine, according to different actions of drugs. E.g., the inhibitor of viral DNA polymerase for NAs, immunomodulator for IFN. We measured the core development period of those drugs by calculating the median year of drug’s patent granted. And by analyzing the patentees, we identified the main contributors for each kind of drug and the changes of their patentee from the first year to the last one (Table 1).

**Table 1:** Patent information of marketed HBV drugs^a^.

Action	Generic name	Median year of patent granted	Patentee and country	First year/ Patentee	last year/ Patentee
Nucleoside analogue (inhibitor of viral DNA polymerase)	Lamivudine	1999	GSK (40%), UK /IAF BioChem (31%), Canada	1990/IAF BioChem, Canada	2010/Emory University, USA
	Adefovir	2004	Gilead Sciences (89%), USA /Bristol-Myers Squibb (11%), USA	1997/ Bristol-Myers Squibb, USA	2011/ Gilead Sciences, USA
	Entecavir	1997	Bristol-Myers Squibb(100%), USA	1993/ Bristol-Myers Squibb, USA	2003/ Bristol-Myers Squibb, USA
	Telbivudine	2005	Novirio/Centre National de la Recherché Scientifique (each 40%), USA	2000/ Centre National de la Recherché Scientifique, USA	2011/ Centre National de la Recherché Scientifique, USA
	Tenofovir	2005	Gilead Sciences (100%), USA	1998/ Gilead Sciences, USA	2014/ Gilead Sciences, USA
Interferon (Immunomodulator)	IFN-alfa (Interferon alfa-2b /Peg interferon alfa-2a)	2004	Schering Corporation (32%), USA Roche (27%), Switzerland	1981/ Biogen, USA	2012/ Schering Corporation, USA
Vaccine		1997	GSK (53%), UK Sanofi(9%), France	1978/ University of Texas System, USA	2013/ GSK, UK

^a^ Marketed HBV drugs contain 3 classes. i.e., nucleoside analogue, interferon, and vaccine. Nucleoside analogs acting as inhibitors of viral DNA polymerase are the main options for current treatment, but none of the drug classes completely eliminates HBV.

**Fig 2:**
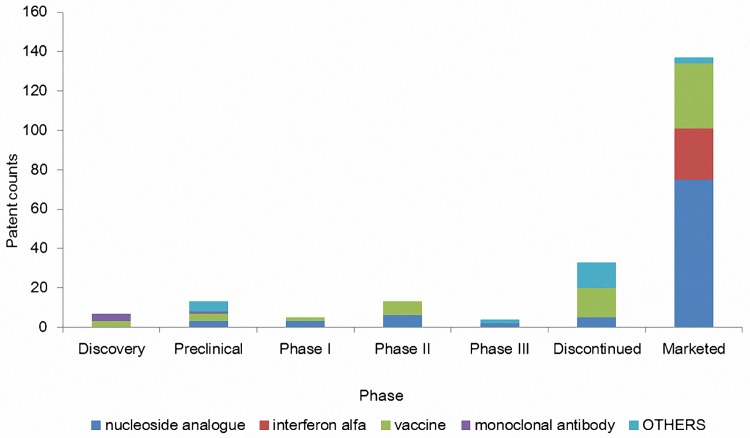
Illustration of distribution of patents by development phase. Note: Most patents are in the marketed phase, indicating stagnancy of R&D of anti-HBV drugs.

**Fig 3:**
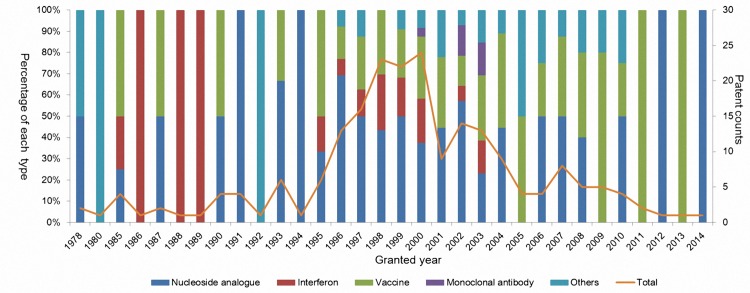
Illustration of distribution of patents by year granted. Note: a reversed U-shaped curve with fluctuations showed the 1995–2004 bloom period of anti-HBV drug development.

**Fig 4:**
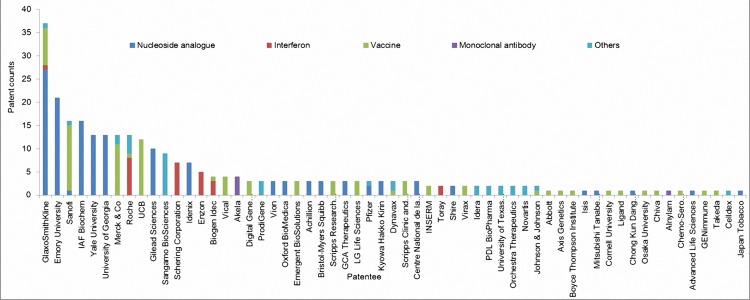
Illustration of distribution of patents by patentee. Note: patentees are listed in descending order from left to right. A portion of the patents are shared by multiple institutions.

A series of diagrams were also produced to display the results of patent distributions. Patent were sorted into different R&D phases including discovery, preclinical, phaseⅠto Ⅲ, and etc (Fig 2). Patents were also classified into years so as to show the temporal changes. And bars with different colors indicate the percentage of each kind of treatment and the orange curve reflects the patent count changes by year (Fig 3). Lastly, patents were assigned to their patentees so as to compare the amount and treatment types of patents held by different patentees (Fig 4).

### Patent citation network analysis

Patent analyses have been utilized in many studies attempting to identify current technology structures and predict technological trends [14] because patents provide detailed technological information and descriptions of the patented innovation [15, 16]. Thus, patents are considered a good proxy for the exchange of and links between technological knowledge, as well as a powerful indicator of the diffusion of technology and the process of improvement. They offer a historical record of the evolution of knowledge and provide a continuous view to view the interactions of technology [12]. Thus, we can trace the genealogy of technological knowledge through patent citations.

As technology systems are highly connected and interdependent, technological structures and linkages are often represented and analyzed in the form of networks. Many studies have integrated patent citations and social networks [17–19]; one recent examples is Stuart et al., who used patents and patent citations to represent a technological network [20]. In other words, patent citation networks can be viewed as a combination of social network theory and bibliometrics methods.

Based on the idea that patent citations can show the relationships between technologies and form specific clusters, we employed social network analysis (SNA) to visualize the technology structures behind the patents and citation information, with the aim of enhancing the identification of both core and emerging technologies in HBV drug development.

In general, a network consists of nodes and links (Alternatively, in social network analysis, they are usually called actors and relationships, respectively). In this study, each node represents a patent and each link with arrow represents the citation. In network analysis, the degree of a node is defined as the number of links or the sum of values of links incident to the node. The in-degree is the number of incoming links to a given node, and it measures technology input and indicates importance in the sense of technological impact. The out-degree represents the citations received by a patent and indicates the importance of the patent in terms of the fundamentality of an invention. Density is a measure of the compactness of networks, defined as the proportion of pairs in a network relative to the total number of pairs possible [21].

The core period of drug evolution in each cluster is reflected by median of the years in which the patents were granted. Moreover, the major patentees of technology input and output in each cluster are determined by their share of in-degree (SI) and out-degree (SO) links. SI is equal to the number of in-degree patents by a patentee divided by the sum of the in-degree patents within a cluster. SO is calculated in the same way using the data of the out-degree on patents. The high SI of a patentee is associated with increased domination in the technology cluster to which they belong.

### Clustering analysis

In network analysis, cluster is also called community or module, and a network community is a sub-network whose nodes are more strongly connected to one another than to the rest of the network [22]. In this study, in order to identify the highly inter-connected nodes in networks, modularity of the partition is used to measure the quality of the partitions and decomposing the networks into sub-units or communities. By running the modularity algorithm integrated into Gephi [23], which is also the tool for visualizing our network model, we obtained multiple resulted clusters for further analysis.

## Results

Consistent with the design of our research framework, we divided the results into three parts and interpreted them following an order of drug information, patent data analysis, and network analysis in order to illustrate the technology flows of patent citation networks for HBV drugs.

### Marketed drugs information

In order to identify the process of technology evolution for HBV drugs and further investigate the related patents of the drugs, we initially collected patent information about marketed HBV drugs from the IMS Patent Focus database and divided the resulting patents into three categories, i.e., nucleoside analogue, interferon, and vaccine. A total of seven anti-HBV therapies are available, as listed in Table 1.

Lamivudine, adefovir, entecavir, telbivudine, and tenofovir are five types of oral NAs that share similar mechanisms by inhibiting viral DNA polymerase and replication. The interferon (IFN) alpha based therapies include two subtypes of immunomodulators, i.e., IFN-α-2b and PEGylated IFN-α-2a. They trigger immune responses and activate antiviral proteins in the human immune system to fight against HBV and therefore can be used either as monotherapy or in combination treatments [10].

Dating back to 1991, IFN-α-2b was the first agent approved for the treatment of HBV infection and its median year of granted patents is 2004. This suggests a long period of 13 years for evaluating IFN. The situation for NAs is quite similar to that of IFN.

In terms of the patent holder, the patentee of Entecavir and Tenofovir remains the same after long time and their patents are still held by Bristol-Myers Squibb and Gilead Sciences, respectively. Oppositely, the owner of patents of lamivudine begins with IAF BioChem and ends up with Emory University. As a well-known and widely-used product from GSK, patents of lamivudine seems to be interested by multiple companies. And a glance at the country of patentee shows that, except for lamivudine, patents related to adefovir, entecavir, telbivudine, and tenofovir are mostly held by American companies or institutions.

### Descriptive statistical analysis

Before the analyses of patent citation data, we initially investigated the distribution of patent data as a view of the distribution of different R&D phases can help us understand the status of drug development. As shown in Fig 2, most patents are in the marketed phase. The data in Fig 2, on one hand, indicate that drug development for HBV infection is approaching a mature stage. On the other hand, it may indicate that new anti-HBV drug development is reaching a bottleneck, which would be worrisome. Patents on IFN cannot be found in any phase but the marketed phase. This suggests the development of IFN has stopped or stagnated. On the contrary, vaccines and NAs seem to have gained more attention from drug developers as they are widely distributed in different development phases.

Fig 3 shows the temporal changes in patent counts. An inverted U-shape with long tails and a peak value in 1998–2000 was captured. There was a sharp increase in patent counts in 1995, with this bloom indicating diversification. Considered the increases in numbers and types of patents, we identified 1995 to 2004 as the bloom period, a golden age of importance for anti-HBV drug development.

The patent counts peak in 2000 when monoclonal antibodies were patented. Since 2004, NAs and vaccines have become the main types of patents, and they now play a dominant role.

Last but not the least, we identified the bloom period of HBV drug development was year 1995–2004 from Fig 3. During this bloom period, the count and diversity of patents increased. Various technology clusters emerged and further increased the patent count. All the median years of clusters are in this bloom period. As close to 65% of patents in this study are at the phase of marketed, and the median years of granting of patent for marketed drugs’ are in this bloom period, we can see a prosperous period for both R&D and commercialization of HBV drugs. In addition, 1978 to 1995 can be viewed as a pre-bloom period and 2005 to 2014 is post-bloom.

In Fig 4, patentees are represented by patenting institution and their patents were counted as long as the institution is associated with a patent, regardless of whether the institution is the only holder of a patent or it shares a patent with other institutions. Obviously, there is an asymmetric distribution of patent counts. GlaxoSmithKline (GSK), a giant pharmaceutical company headquartered in London, leads in patent counts and has investigated many different kinds of therapies.

From the perspective of technology diversity, patentees can be divided into two classes. The first class includes GSK, Sanofi, and Roche. They make up a technologically diversified class because they possess multiple technologies for HBV infection treatments. The second class of patentee focuses on a single technology, such as Emory University, IAF BioChem, and Yale University.

### Patent citation network

#### Overall profile

**Fig 5:**
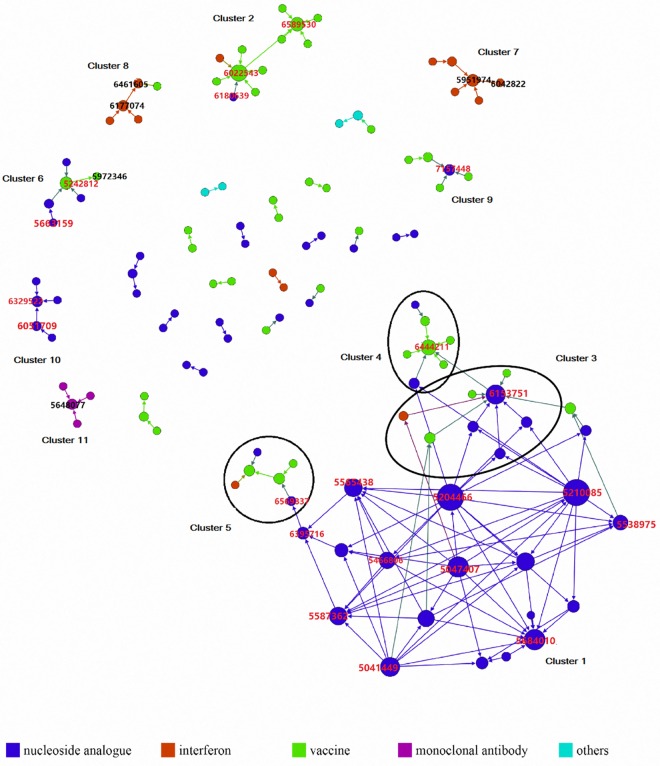
Patent citation network. Note: Network model is divided into different clusters and patents worth noticing are annotated with their US patent numbers (the prefix “US” is removed and only the numbers are shown for users to search in the USPTO’s online database).

To identify the technological flow of HBV drugs, a patent citation network was generated based on the citation information from the 212 patents recorded in the USPTO database; nodes and arrows represent patents and citations, respectively, and are colored based on different types of treatments (Fig 5).

As shown in the patent citation network (Fig 5), there are 122 nodes representing patents and 146 edges representing citations. Ninety nodes were removed as they were not linked with any other nodes. The average degree of the whole network is 1.197, calculated by dividing the sum of all node degrees by the total count of nodes in the network. The degree of a node can be calculated as the number of links that a given node has to other nodes. The patent citation network is a directed network, i.e., the direction of a link is determined by the citation relationship: citing or cited [24]. Thus, the in-degree and out-degree are defined as different degrees. The in-degree represents the number of times a patent cites other patents. This reflects the adoption of technology from former patents by a newer patent and therefore can be used to measure technology input. The out-degree represents the number of times a patent is cited by other patents, which measures the technology output [13].

#### Comparison among clusters

To analyze the technology flow and community, the network has been divided into several clusters through the fast unfolding modularity algorithm as described in the methodology section [22]. Furthermore, the R&D statuses, patentees, and network topological features of resulted 11 clusters were further analyzed and are summarized into Table 2. Specifically, the average degree of a cluster can be defined as the average number of links between nodes, which can be used to identify the tightness of the interactive relation within a cluster. E.g., cluster 1 dominated by NAs shows a much higher average degree than the other clusters. Based on this attribute, NAs show a more interconnected structure and closer interaction in technology flows.

**Table 2:** Information of the main technology clusters in the patent citation network^a^.

Cluster numbers	Nodes	Edge	Average degree	Density	Main type	Phase	Median year of patent granted	Patentee with largest SI ^b^	Patentee with largest SO ^C^
Cluster 1	19	57	3.00	0.17	Nucleoside analogue	Marketed	1996	University of Georgia (39%)	Emory University (31%)
Cluster 2	12	11	0.92	0.08	Vaccine	Marketed/ Discontinued	1996	UCB (100%)	UCB (38%)
Cluster 3	8	7	0.88	0.13	Nucleoside analogue	Marketed	1998	Emory University /GlaxoSmithKline /IAF Biochem (30%of each)	IAF Biochem(44%)
Cluster 4	7	6	0.86	0.14	Vaccine	Marketed/ Discontinued	2004	Sanofi (78%)	Sanofi (43%)
Cluster 5	6	5	0.83	0.17	Vaccine	Marketed	2003	GlaxoSmithKline (83%)	Centre National de la Recherche Scientifique /GlaxoSmithKline /Novirio (29% of each)
Cluster 6	6	5	0.83	0.17	Nucleoside analogue	Marketed/ Phase II	2003	LG Life Sciences (80%)	Bristol-Myers Squibb (40%)
Cluster 7	6	5	0.83	0.17	Interferon	Marketed	1998	Enzon (100%)	Enzon (60%)
Cluster 8	5	4	0.80	0.20	Vaccine	Marketed/ Preclinical	1998	Schering Corporation (100%)	Schering Corporation (50%)
Cluster 9	5	4	0.80	0.20	Nucleoside analogue	Marketed	1997	GlaxoSmithKline (75%)	Sanofi(50%)
Cluster 10	5	4	0.80	0.20	Nucleoside analogue	Marketed	1999	GlaxoSmithKline (100%)	GlaxoSmithKline (50%)
Cluster 11	4	3	0.75	0.25	Monoclonal antibody	Discovery	2003	PDL BioPharma (100%)	PDL BioPharma (100%)

^a^ Clusters representing different technology communities, their topological parameters in the network model, and related patent information are listed.

^b^ SI: Share of in-degree. SI is equal to the number of in-degree patents by a patentee divided by the sum of the in-degree patents within a cluster.

^c^ SO: Share of out-degree. SO is equal to the number of out-degree patents by a patentee divided by the sum of the out-degree patents within a cluster.

A general look at Fig 5 tells that, cluster 1 is the biggest cluster in the overall network and is highly interactive, indicating on-going development of NAs. The interconnecting clusters 1, 3, 4, and 5 can be further combined into a big component including the technologies of NAs, interferon, and vaccines. Similarly, clusters 2, 6, 8, and 9 also display nodes with different colors and technology combinations through patent citations. The rest of clusters, i.e., clusters 7, 10, and 11, are dominated by only a single type of treatment and can be viewed as technologically concentrated community. E.g., cluster 11 is dominated by merely the single technology of monoclonal antibodies. And from the perspective of network, the technology of monoclonal antibody has few connections with other technologies and therefore cluster 11 belongs to the type of technologically concentrated community. The same situation can be found in cluster 10 as well. Interestingly, cluster 11 is the only cluster about monoclonal antibody treatment and it consists of 4 patents all held by PDL BioPharma only (Table 2). While a point to notice is that, the therapy of monoclonal antibody is still under investigation and thus lots of works are to be done to address issues about clinical efficacy and safety.

Moreover, it has been observed in Fig 5 that, within cluster 1 to 6, vaccine patents lie in the end or terminal points of technology flows, and vaccines always appear together with other technologies in clusters. This is an important discovery indicating vaccines may play a role of technological synthesizer in anti-HBV drug development. This provides researchers with insights for future R&D on anti-HBV drugs. Take cluster 5 as an example, patent US6013264 located at the end of the technology flow is vaccine composition technology comprising HBV surface antigens. Similarly, according to the patent claim, US5972346 at the end of cluster 6 is a kind of therapeutic vaccine used in medical treatment for on-going hepatitis viral infections.

## Discussion

Patent citations have been used to represent technology transfer or technology spillover in many studies [25], and it has been regarded as a good measurement of technology flows among different industries and fields of technology [26]. In this study, we carried out systematic analyses of patent data on anti-HBV drugs in order to identify its technology flow. We presented the results of patent distribution, network analysis, and cluster comparison analysis in order to gain a deeper understanding of the technological knowledge flows in the development of anti-HBV drugs. We selected network analysis as our core method for patent citation analysis because citations can be modeled using arrows to measure the direction of technology flow in HBV drug development.

The reported results consist of three parts, each of which emphasized a unique aspect of anti-HBV drug patents. Firstly, analyses of marketed anti-HBV drugs from IMS databases identified the U.S.A. is enjoying technological advantages in anti-HBV drug development. Among drugs available on the market, entecavir and tenofovir outperform their competitors because of their relatively high potency and low resistance profile [27, 28].

Secondly, a diagram of patent distribution (Fig 2) showed that patents of NAs have the advantage of other drugs regarding patent count. In addition to the large quantity, they have the characteristics of high interactions and continual development in the technological community (Fig 5). Likewise, NAs administered orally are more suitable for patients owing to its potent antiviral activity along with fewer side effects [28]. Further, in consideration of the efficacy and safety of drugs, NAs has been positioned as the current mainstream treatment of HBV infection [29]. Whilst Fig 2 of distribution of development phase showed that most patents are located in marketed drugs, suggesting stagnancy of the research and development of anti-HBV drugs. This conclusion also supports by Fig 5 and the phenomenon is quite different from our previous reports of patent studies of anti-Alzheimer's drugs, therapeutic monoclonal antibodies, and dendritic cells [13, 30, 31]. In our previous reports, the patents were mainly distributed in the earlier phases of clinical trials; such distribution patterns indicate the on-going processes of R&D.

What is more, we found the increased diversity of the cooperation form between entities, which affects the R&D of anti-HBV drugs. Conventionally, university–industry collaborations in biotech industry have been common [32]. For instance, through careful examination on patent documents, we identified university-industry cooperation cases amongst the top 10 anti-HBV drug patentees in Fig 4, (Here, we consider that, cooperation exists among patentees if a patent is held by two or more patentees from different institutions.), i.e., Emory University, Yale University, University of Georgia, and seven pharmaceuticals [33].

Whilst the conventional university-industry cooperation is the predominant form, the Bayh–Dole Act passed in 1980 has increased the diversity of cooperation forms. The Act in fact benefits both the industrial corporations and the public research institutions it not only greatly facilitated the technology transfer by industrial corporations, but also stimulated the cooperation mode by encouraging universities to commercialize the federally funded research projects [33, 34]. In the other word, the Bayh-Dole Act boosted the university–university cooperation as it allows the patentee identity of universities in the patenting system. For the academic institutions, the Bayh-Dole Act facilitated the growth of university patenting and licensing of technologies [33]. Specifically, in 2008, American universities have owned licensing revenues of $3.4 billion, as opposed to $7.3 million in 1981 [35]. Moreover, revenue from the commercialization of technology becomes an increasingly important and substantial source of financial support for universities in the United States, with combined revenues from licensing and industry-supported research in all fields reaching well over $6 billion per year [34].

Here, we take Emory University as the typical example for illustrating the diversity of cooperation relationship. According to our analysis, Emory University possesses 21 (38.1% of the total 55 patents held by two or more institutions) patents shared with other patentees. Emory University shares these patents with both academic and industrial partners such as GSK, University of Georgia, Gilead Sciences, Japan Tobacco, end etc. Another example of university–university collaboration is the University of Georgia, holding 13 patents in nucleoside analogues as co-owner with Emory University (4/13) and Yale University (9/13). These cases show that, university-university cooperation is the novel types of patentee form in addition to the conventional university–industry cooperation.

Inspired by cases above, we suggest policymakers should act on policies so as to encourage diverse forms of cooperation in the R&D community. For domestic pharmaceutical companies in developing countries, which are facing severe HBV infection threats, we suggest building cooperative relationships with large pharmaceutical companies possessing advanced anti-HBV technologies, since the health of its population is a vital issue to a country. It is also possible and beneficial for local governments to play a coordinating role in such international forms of cooperation.

Thirdly, through the patent citation network and cluster analysis, technology flow and technology-based R&D communities were identified. One of our most interesting discoveries is that, the overall network model of ours shows high dispersion and most of clusters are completely separated and have little contact with each other (Fig 5). As mentioned previously, patent citations represent a technological connection. Therefore, this indicates that the technologies of HBV drugs have less interaction with each other than technologies investigated in our previous reports [13, 30, 31], where network models were highly connected and exhibited patterns of continuous growth and expansion. Hence, we conclude that the features of the network model of anti-HBV drugs display less potential in further growth and expansion. This is graphically consistent with what we found in the patent distribution (Fig 2), i.e., anti-HBV drug development has reached a bottleneck.

Another interesting discoveries is the multi-technological concentricity of vaccines (Fig 5). The technology flow of anti-HBV drug development identified by our network analysis suggests that vaccine technologies adopt the knowledge from patents of NAs and INF drugs, and hence the vaccine technologies are the potential next generation therapy for HBV infection treatment.

A study showed that the combination of vaccines with immunotherapy or classical antiviral treatments may be a more effective treatment strategy due to additive or synergistic efficacy, and this kind of vaccines can be viewed as therapeutic vaccines [36]. Inspired by this, we searched and listed a part of clinical trials for therapeutic vaccines and immunomodulatory agents against HBV infection in Table 3. In order to access the safety of the novel technologies, we further investigated the safety reports of these trials. To our disappointment, few information of side effect and adverse drug reaction is available so far. And amongst the trials, HB-110 from Genexine, Inc., a kind of novel therapeutic DNA vaccine against chronic hepatitis B, is currently in phaseⅠand a study from Yoon et al. [37] indicated that HB-110 is potentially safe and tolerable in CHB patients. This is also supported by data from IMS R&D Focus database and other clinical trials with similar safety comment are DV-601 and ppdpSC18 in Table 3.

**Table 3:** Partial list of current clinical trials evaluating various vaccine therapies for HBV^a^^,^ ^b^.

Trial	Phase	Interventions	Drugs	Sponsor/Collaborators
NCT02505009	Phase Ⅳ	Engerix-B; Entecavir; Tenofovir	Engerix-B	Chang Gung Memorial Hospital
NCT02360592	Phase Ⅳ	Entecavir; IFN alfa-2b; Interleukin 2; Hepatitis B Vaccine	IFN + Interleukin 2 + Vaccine	Tongji Hospital
NCT02097004	Phase Ⅳ	Peg-IFN alfa-2a; HBV vaccination; Entecavir	Therapeutic Vaccination + Peg- IFN	Seoul National University Hospital
NCT00120796	Phase Ⅲ	Lamivudine; Recombinant hepatitis B surface antigen	Lamivudine + Therapeutic Vaccine	French National Agency for Research on AIDS and Viral Hepatitis /GlaxoSmithKline
NCT02249988	Phase Ⅲ	ABX203 therapeutic Hepatitis B vaccine	ABX203	Abivax S.A.
NCT01374308	Phase Ⅲ	NASVAC; Pegylated IFN alpha 2b	NASVAC	Clinical Research Organization, Dhaka, Bangladesh
NCT02615639	Phase Ⅱ	HPDC-T cells;IFN-a-2a;Telbivudine;Entecavir	HPDC-T cells + Entecavir	Third Affiliated Hospital of Sun Yat-Sen University
NCT02693652	Phase Ⅱ	CVI-HBV-002	CVI-HBV-002	CHA Vaccine Institute Co., Ltd.
NCT00536627	Phase Ⅱ	DNA vaccine pCMVS2.S	pCMVS2.S	French National Agency for Research on AIDS and Viral Hepatitis
NCT01023230	Phase Ⅰ	DV-601; Entecavir	DV-601	Dynavax Technologies Corporation
NCT01817725	Phase Ⅰ	HBV vaccine (Engerix B)	Engerix B	Chang Gung Memorial Hospital
NCT01641536	Phase Ⅰ	HB-110	HB-110	Genexine, Inc.
NCT00988767	Phase Ⅰ	pCMV-S2.S DNA (DNA vaccine)	pCMV-S2.S	Institut National de la Santé Et de la Recherche Médicale, France
NCT00277576	Phase Ⅰ	ppdpSC18	ppdpSC18	PowderMed
NCT02496897	Phase Ⅰ	FP-02.2 Vaccine; Placebo;IC31® Adjuvant	FP-02.2 Vaccine	Altimmune, Inc.
NCT00513968	Phase Ⅰ	HB-110; Adefovir	HB-110	Genexine, Inc.
NCT01813487	Unknown	HBsAg vaccine+ Entecavir	HBsAg vaccine with Entecavir	Genexine; Inc.

^a^ List of clinical trials of potential novel drugs for HBV infection treatment. Data are from www.clinicaltirals.gov.

^b^ Safety comments data are from IMS R&D Focus and www.clinicaltirals.gov.

Last but not least, results of our study are insightful and we would like to share the inspirations with different groups of people including researcher, investors and governmental officers.

First and foremost, in terms of currently available therapies for HBV infection, there are some points to be improved. One example is the failure to completely eliminate HBV and the other is the poor availability of drugs due to high expense, especially for developing countries whose infected populations are quite large. China, estimated to have an infected population of 93 million [9] and with one of the highest rates of HBV infection in the world, should actively engage, together with other developing countries, in the fight against HBV infection. In China, the HBV vaccination was integrated into an expanded program of immunization vaccines by the government in 2001, but the service fee for the vaccination procedure was still charged to those families. It was not until 2005 that the Chinese government adopted a completely free HBV vaccination program for all neonates. This means those older than 10 years may not be protected by the vaccination program, while this part of the unprotected and infected population also serves as the primary labor force in China’s economy. Thus, developing effective drugs for this large segment of the population is an urgent mission.

Next, from the perspective of drug development, there is a great demand for new drug development because limitations, such as failure of elimination of HBV and long-term treatment, still exist in current therapies [30]. As explained in the previous section, current drugs mainly act as inhibitors by targeting HBV replication, while they are unable to eliminate HBsAg. The following two points may account for this technological bottleneck. Firstly, pharmaceutical companies may have decreasing interest in anti-HBV drug development due to the high risk of failure and the opportunity cost, i.e., the huge investments and lengthy periods required for drug development. Secondly, pharmaceutical companies have become less interested in developing novel anti-HBV treatments due to the lower prevalence of HBV infection in developed countries, as well as the fact that from an investment standpoint, new drugs that might cure people from HBV infection may not bring as much profit as current drugs that require long-term therapy.

Despite these negative circumstances, there is still hope of fighting against HBV infection if we are willing to undertake the challenge. The government of China is now fully aware of the importance of drug innovation and is investing large amount of capital into it. As a result, several large drug innovation projects initiated and led by the Chinese Academy of Science were launched in recent decades. On one hand, we are glad to see the efforts and ambition of the Chinese research communities. On the other hand, we are concerned about the priority of these projects and suggest that drug innovation for HBV infection should be given a high priority to cater to the urgent needs of HBV infection treatments.

The SI and SO of clusters, which were introduced in previous section, also offer strategic hints for anti-HBV drug development in China, Theoretically, the out-degree indicating novel technological innovation presents the competitive strength and is more important than the in-degree of patent nodes representing the adoption or absorption of existed technologies. Unfortunately, current pipeline for hepatitis B drug development is drying up, and therefore it is difficult to achieve such out-degree breakthrough or innovation. This is also true in China, and the in-degree way is now the majority of hepatitis B drug development in China. Therefore, the institutions with the larger SI can be viewed as potential partner to cooperate with.

Although there is a great demand for new anti-HBV drugs, we fully understand the difficulties of developing a novel therapy with better efficacy and cheaper price. Fortunately, our technology flow analysis indicates that vaccine may be the next generation therapy and therefore is one of the potential ways for researchers and investors to go.

Despite our encouragement of the international cooperation, a country should ultimately construct an integrative system or environment of its own to facilitate the development of pharmaceutical technologies. The US is a good example and has been doing this very well. The effective communication and smooth cooperation among academic researchers, industries, and governmental organizations in the US give it a leading role in R&D in the global pharmaceutical market. In summary, novel drug development is a large project and good cooperation and coordination among governmental organizations, university researchers, and pharmaceutical companies are of vital importance to the development and success of new drugs.

Last but not the least, our study is with space for improvement as well. First and foremost, as every coin has two sides, in terms of our core methodology, i.e., the patent citation network analysis, which heavily relies on patents and their citation information, has the following potential drawbacks, too. One is that it is difficult to guarantee and expect all technological innovations can be found in the patent pools, as applying patents is quite a kind of subjective, time- and resource-consuming behavior. More straightforward, small and middle-sized enterprises (SMEs) with less resources may not be so active as giant corporations in patent filing. The next point is, patents may not contain R&Ds carried out by some public sectors, especially who are not targeting the translational researches. Generally speaking, these sectors lack incentives and external stimulations for patent filing. In such aspect, patent citation network may not be able to completely reflect the R&D tracks as well as the technology flow. The second pity is that, we could not provide more details about the safety information of the aforementioned clinical trials, which prevents the deeper evaluation of technologies involved. The last point is, we also tried to tell the therapeutic vaccine-related patents from the preventive vaccine-related patents, as these two kinds of vaccine are in different purposes for use, however, most of the relevant patent claims obtained in this study do not contain information for telling the preventive and therapeutic vaccine apart. Thus, we are not able to further specify the type of vaccine in this study.

Initially, in this study, we investigated the marketed anti-HBV drugs and analyzed their technologies. With descriptive statistical analysis on their corresponding patent data, we identified the stagnant status of anti-HBV drug development and pointed the way for development of domestic pharmaceuticals in developing countries. Next, our analysis on patentee data discovered that, the novel cooperation forms added diversity to the conventional form of cooperation within the biotech and pharmaceutical industry. Last but not the least, we depicted an overall network model so as to visualize and then analyze the whole technology community of anti-HBV drug development, in which we also discussed about therapeutic vaccines as the potential next generation therapy for HBV infection. Our work thoroughly provides inspiring insights for investors, policy makers, researchers, and other readers interested in anti-HBV drug development.

## Supporting Information

S1 File Search criteria. A series of criteria for searching against IMS LifeCycle database were included in the S1 File.
(DOCX)

S2 File 212patents_list. The detailed information of the resultant 212 patents was listed in the file.
(XLSX)

## References

[1] WHO. Guidelines for the prevention, care and treatment of persons with chronic hepatitis B infection WHO Press 2015. 26225396

[2] Lai CL, Ratziu V, Yuen M-F, Poynard T. Viral hepatitis B. The Lancet. 2003;362(9401):2089–94. 10.1016/s0140-6736(03)15108-2 14697813

[3] Liaw YF. Reduction of cirrhosis and hepatocellular carcinoma with antiviral therapy in chronic hepatitis B. Hepatology. 2013;58(5):1856 10.1002/hep.26358 . 23463378

[4] Lok AS-F. Hepatitis: Long-term therapy of chronic hepatitis B reverses cirrhosis. Nat Rev Gastroenterol Hepatol. 2013;10(4):199–200. 10.1038/nrgastro.2013.13 23358397

[5] Lozano R, Naghavi M, Foreman K, Lim S, Shibuya K, Aboyans V, et al Global and regional mortality from 235 causes of death for 20 age groups in 1990 and 2010: a systematic analysis for the Global Burden of Disease Study 2010. Lancet. 2012;380(9859):2095–128. 10.1016/S0140-6736(12)61728-0 . 23245604 PMC10790329

[6] Sun J, Hou JL. Management of chronic hepatitis B: experience from China. J Viral Hepat. 2010;17 Suppl 1:10–7. 10.1111/j.1365-2893.2010.01274.x . 20586929

[7] Buchmann P, Dembek C, Kuklick L, Jager C, Tedjokusumo R, von Freyend MJ, et al A novel therapeutic hepatitis B vaccine induces cellular and humoral immune responses and breaks tolerance in hepatitis B virus (HBV) transgenic mice. Vaccine. 2013;31(8):1197–203. 10.1016/j.vaccine.2012.12.074 . 23306359

[8] Barnes E. Therapeutic vaccines in HBV: lessons from HCV. Med Microbiol Immunol. 2015;204(1):79–86. 10.1007/s00430-014-0376-8 25573348 PMC4305103

[9] Cox N, Tillmann H. Emerging pipeline drugs for hepatitis B infection. Expert Opin Emerg Drugs. 2011;16(4):713–29. 10.1517/14728214.2011.646260 . 22195605

[10] Nunamaker JF, Li J, Zhang Z, Chen H, Li X. Managing Knowledge in Light of Its Evolution Process: An Empirical Study on Citation Network-Based Patent Classification. Journal of Management Information Systems. 2009;26(1):129–54. 10.2753/mis0742-1222260106

[11] Jaffe AB, Trajtenberg M. International Knowledge Flows: Evidence From Patent Citations. Economics of Innovation and New Technology. 2006;8(1–2):105–36. 10.1080/10438599900000006

[12] Sorenson O, Rivkin JW, Fleming L. Complexity, networks and knowledge flow. Research Policy. 2006;35(7):994–1017. 10.1016/j.respol.2006.05.002 .

[13] Xu J, Kong X, Qiu L, Geng X, Hu Y, Wang Y. Research and development of anti-Alzheimer's drugs: an analysis based on technology flows measured by patent citations. Expert Opin Ther Pat. 2014;24(7):791–800. 10.1517/13543776.2014.915943 . 24798577

[14] Cho TS, Shih HY. Patent citation network analysis of core and emerging technologies in Taiwan: 1997–2008. Scientometrics. 2011;89(3):795–811. 10.1007/s11192-011-0457-z .

[15] Park H, Yoon J. Assessing coreness and intermediarity of technology sectors using patent co-classification analysis: the case of Korean national R&D. Scientometrics. 2013;98(2):853–90. 10.1007/s11192-013-1109-2

[16] Verspagen B. Mapping technological trajectories as patent citation networks: A study on the history of fuel cell research. Advances In Complex Systems. 2007;10(1):93–115. 10.1142/S0219525907000945 .

[17] Chang SB, Lai KK, Chang SM. Exploring technology diffusion and classification of business methods: Using the patent citation network. Technological Forecasting And Social Change. 2009;76(1):107–17. 10.1016/j.techfore.2008.03.014 .

[18] Li X, Chen H, Huang Z, Roco MC. Patent citation network in nanotechnology (1976–2004). Journal of Nanoparticle Research. 2007;9(3):337–52. 10.1007/s11051-006-9194-2

[19] Gress B. Properties of the USPTO patent citation network: 1963–2002. World Patent Information. 2010;32(1):3–21. 10.1016/j.wpi.2009.05.005

[20] Stuart TE, Podolny JM. Local search and the evolution of technological capabilities. Strategic Manage J. 1996;17:21–38. 10.1002/smj.4250171004 .

[21] Yoon J, Kim K. Identifying rapidly evolving technological trends for R&D planning using SAO-based semantic patent networks. Scientometrics. 2011;88(1):213–28. 10.1007/s11192-011-0383-0

[22] Blondel VD, Guillaume JL, Lambiotte R, Lefebvre E. Fast unfolding of communities in large networks. J Stat Mech-Theory E. 2008 Artn P10008 10.1088/1742-5468/2008/10/P10008. 10.1088/1742-5468/2008/10/p10008 .

[23] Jacomy M, Venturini T, Heymann S, Bastian M. ForceAtlas2, a continuous graph layout algorithm for handy network visualization designed for the Gephi software. PLoS One. 2014;9(6):e98679 10.1371/journal.pone.0098679 24914678 PMC4051631

[24] Choe H, Lee DH, Seo IW, Kim HD. Patent citation network analysis for the domain of organic photovoltaic cells: Country, institution, and technology field. Renewable and Sustainable Energy Reviews. 2013;26:492–505. 10.1016/j.rser.2013.05.037

[25] Stolpe M. Determinants of knowledge diffusion as evidenced in patent data: the case of liquid crystal display technology. Research Policy. 2002;31(7):1181–98. Pii S0048-7333(01)00192-5 10.1016/s0048-7333(01)00192-5

[26] Karki MMS. Patent citation analysis: A policy analysis tool. World Patent Information. 1997;19(4):269–72. 10.1016/s0172-2190(97)00033-1

[27] Yuen MF, Lai CL. Treatment of chronic hepatitis B: Evolution over two decades. J Gastroenterol Hepatol. 2011;26 Suppl 1:138–43. 10.1111/j.1440-1746.2010.06545.x . 21199525

[28] Locarnini S, Hatzakis A, Chen DS, Lok A. Strategies to control hepatitis B: Public policy, epidemiology, vaccine and drugs. J Hepatol. 2015;62(1S):S76–S86. 10.1016/j.jhep.2015.01.018 . 25920093

[29] Yuen MF. Anti-viral therapy in hepatitis B virus reactivation with acute-on-chronic liver failure. Hepatol Int. 2015;9(3):373–7. 10.1007/s12072-014-9569-x . 25788180

[30] Kong X, Hu Y, Cai Z, Yang F, Zhang Q. Dendritic-cell-based technology landscape: Insights from patents and citation networks. Hum Vaccin Immunother. 2015;11(3):682–8. 10.1080/21645515.2015.1008857 25714961 PMC4514221

[31] Geng X, Kong X, Hu H, Chen J, Yang F, Liang H, et al Research and development of therapeutic mAbs: An analysis based on pipeline projects. Human Vaccines & Immunotherapeutics. 2015;11(12):2769–76. 10.1080/21645515.2015.1074362 26211701 PMC4916486

[32] Smith HL, Bagchi‐Sen S. University–Industry Interactions: the Case of the UK Biotech Industry. Industry & Innovation. 2006;13(4):371–92. 10.1080/13662710601032697

[33] Mowery DC, Nelson RR, Sampat BN, Ziedonis AA. The growth of patenting and licensing by U.S. universities: an assessment of the effects of the Bayh–Dole act of 1980. Research Policy. 2001;30(1):99–119. 10.1016/s0048-7333(99)00100-6

[34] Drozdoff V, Fairbairn D. Licensing biotech intellectual property in university-industry partnerships. Cold Spring Harb Perspect Med. 2015;5(3):a021014 10.1101/cshperspect.a021014 . 10.1016/S0048-7333(01)00192-5. WOS:000176356200009. 25605752 PMC4355252

[35] Loise V, Stevens AJ. The Bayh-Dole Act Turns 30. Science Translational Medicine. 2010;2(52):52cm27–52cm27. 10.1126/scitranslmed.3001481 20926832

[36] Michel ML, Deng Q, Mancini-Bourgine M. Therapeutic vaccines and immune-based therapies for the treatment of chronic hepatitis B: perspectives and challenges. J Hepatol. 2011;54(6):1286–96. 10.1016/j.jhep.2010.12.031 . 21238516

[37] Yoon SK, Seo YB, Im SJ, Bae SH, Song MJ, You CR, et al Safety and immunogenicity of therapeutic DNA vaccine with antiviral drug in chronic HBV patients and its immunogenicity in mice. Liver International. 2015;35(3):805–15. 10.1111/liv.12530 24620920

