# Supplementary material for: Research and Development of Hepatitis B Drugs: An Analysis Based on Technology Flows Measured by Patent Citations
Source: PLoS One. 2016 Oct 11;11(10):e0164328. doi: 10.1371/journal.pone.0164328 (PMC5058496; doi:10.1371/journal.pone.0164328)
Supplement: S1 File — A series of criteria for searching against IMS LifeCycle database were included in the S1 File. (DOCX) [file pone.0164328.s001.docx]

DATA searching criteria between year 1975~2015

This study collected data from the IMS LifeCycle databases, which is a collection of databases about pharmaceuticals.

In the sub-database of IMS LifeCycle, i.e., patent focus and R&D focus, we utilized the keyword of “hepatitis b” in the search filed of “indication”, so as to obtain relevant patent information.

The original patents retrieved were sorted according to their country of origin, and all of patents were transformed into the corresponding US patent format via the patent family system of the European Patent Office (EPO) because analyzing and comparing patent data using one single patent system results in more standard, comparable, and unified patent citation information.
